# Implementation and Outcomes of a Collaborative Multi-Center Network Aimed at Web-Based Cognitive Training – COGWEB Network

**DOI:** 10.2196/mental.3840

**Published:** 2014-11-27

**Authors:** Vítor Tedim Cruz, Joana Pais, Luis Ruano, Cátia Mateus, Márcio Colunas, Ivânia Alves, Rui Barreto, Eduardo Conde, Andreia Sousa, Isabel Araújo, Virgílio Bento, Paula Coutinho, Nelson Rocha, COGWEB Network Collaborators

**Affiliations:** ^1^ Hospital São Sebastião, Centro Hospitalar Entre Douro e Vouga Neurology Department Santa Maria da Feira Portugal; ^2^ Clinical Research Office Health Sciences Department University of Aveiro Aveiro Portugal; ^3^ Hospital São Sebastião, Centro Hospitalar Entre Douro e Vouga Neuropsychology Laboratory, Neurology Department Santa Maria da Feira Portugal; ^4^ Public Health Institute - ISPUP University of Porto Porto Portugal; ^5^ University Institute of Maia - ISMAI Maia Portugal; ^6^ UnIGENe Instituto de Biologia Molecular e Celular University of Porto Porto Portugal; ^7^ COGWEB network Lisbon Portugal

**Keywords:** cognitive training, neurorehabilitation, eHealth systems, memory clinic, collaborative network, stroke, dementia, schizophrenia, mental health services

## Abstract

**Background:**

Cognitive care for the most prevalent neurologic and psychiatric conditions will only improve through the implementation of new sustainable approaches. Innovative cognitive training methodologies and collaborative professional networks are necessary evolutions in the mental health sector.

**Objective:**

The objective of the study was to describe the implementation process and early outcomes of a nationwide multi-organizational network supported on a Web-based cognitive training system (COGWEB).

**Methods:**

The setting for network implementation was the Portuguese mental health system and the hospital-, academic-, community-based institutions and professionals providing cognitive training. The network started in August 2012, with 16 centers, and was monitored until September 2013 (inclusions were open). After onsite training, all were allowed to use COGWEB in their clinical or research activities. For supervision and maintenance were implemented newsletters, questionnaires, visits and webinars. The following outcomes were prospectively measured: (1) number, (2) type, (3) time to start, and (4) activity state of centers; age, gender, level of education, and medical diagnosis of patients enrolled.

**Results:**

The network included 68 professionals from 41 centers, (33/41) 80% clinical, (8/41) 19% nonclinical. A total of 298 patients received cognitive training; 45.3% (n=135) female, mean age 54.4 years (SD 18.7), mean educational level 9.8 years (SD 4.8). The number enrolled each month increased significantly (*r*=0.6; *P=*.031). At 12 months, 205 remained on treatment. The major causes of cognitive impairment were: (1) neurodegenerative (115/298, 38.6%), (2) structural brain lesions (63/298, 21.1%), (3) autoimmune (40/298, 13.4%), (4) schizophrenia (30/298, 10.1%), and (5) others (50/298, 16.8%). The comparison of the patient profiles, promoter versus all other clinical centers, showed significant increases in the diversity of causes and spectrums of ages and education.

**Conclusions:**

Over its first year, there was a major increase in the number of new centers and professionals, as well as of the clinical diversity of patients treated. The consolidation of such a national collaborative network represents an innovative step in mental health care evolution. Furthermore, it may contribute to translational processes in the field of cognitive training and reduce disease burden.

## Introduction

### Professional Collaborative Networks and Cognition Care

The evolution of health systems is increasingly dependent on professional collaborative networks [1,2]. This type of solution has been thoroughly explored in social, governmental, commercial, and enterprise competitive settings [3,4]. Nonetheless, in the health care setting, there is a limited understanding of the network dynamics, internal processes, key structural features, or how to evaluate their outcomes [5-7].

In general, professionals see collaboration as necessary, and their main expectations are to establish interprofessional relations that would lead to greater efficiency, better knowledge of other institutions, and professional support [8]. However, most health care settings are prone to generate isolated clusters, like professional groups, medical specialties, organization departments, and units [9]. They usually are kept apart due to physical, cultural, cognitive, or trust barriers [10].

The mental health sector, mainly due to demographic and economic constraints on health resources, is under increasing pressure to self-reshape and implement new sustainable approaches [11-13]. This situation has been enlightening groups and key players, at several hierarchic levels of decision, to the advantages of working together in search of synergies and more effective ways to deliver mental care [2,11,14].

Cognitive deficits associated with the most prevalent neurologic and psychiatric diseases represent 11.2% of the global burden of disease worldwide, accounting each year for 30 new cases per 1000 inhabitants [15]. Nowadays, treatment of cognitive deficits largely relies on specialized human mediated interventions (eg, cognitive rehabilitation, training, stimulation, or remediation), with pharmacological options far from playing an important role [16]. The combination of these factors renders most mental health systems worldwide largely unable to meet cognitive rehabilitation needs, either in due time after injury or adequate intensities [2]. To adequately meet these new demand patterns without increasing health care costs, sustainable organizational changes are necessary [2,17]. In addition, the clinical use of information technology based systems is known to improve cognitive interventions, namely their intensity, patient adherence, and quality of professional monitoring [18-21].

### An Innovative Web-Based Cognitive Training System

With this global scenery in mind, starting in 2005 in a memory clinic setting, we developed an innovative Web-based cognitive training system, named COGWEB and described elsewhere [22-24]. Over time, the system evolved to address the needs of patients, professionals, and organizations in the field of cognitive rehabilitation [22,25]. It was designed to: (1) improve the efficiency of home-based cognitive training procedures; (2) increase patient access to care; (3) shift the therapeutic footprint from hospital to patient comfort zones; and most importantly, (4) to foster collaborative work between professionals from geographically distributed centers [24,25]. This set of characteristics made the COGWEB system especially suited to be the promoter of a new collaborative network, sharing specialized knowledge, improved procedures, innovative tools, and connecting professionals and institutions dedicated to cognitive rehabilitation.

The aim of this paper is to describe the implementation, early outcomes, and sustainability, over its first year of functioning, of a nationwide multi-organizational cognitive interventional network, taking advantage of the characteristics of an innovative Web-based cognitive training system.

## Methods

### National Setting

#### Cognitive Interventions

The Portuguese mental health sector has some specificities [26], nevertheless most of its organization is comparable to Western European models of care [15,27]. Neuropsychological rehabilitation is performed in different and almost unrelated settings in Portugal [28]. If we consider all forms of cognitive intervention provided (rehabilitation, training, stimulation, or remediation) along mental health services, as defined by the World Health Organization [15,27] and the National mental health plan [26,29], we may group them in the following ways.

#### Referral Institutions With Medical Supervision or Integrated in Multi-disciplinary Clinical Departments

The adult outpatient memory clinics in neurology and psychiatry departments are mainly dedicated to neuropsychological assessment, but some of them are also interested in providing rehabilitation care.

The day centers within psychiatric clinics and departments are dedicated to patients with schizophrenia, major depression, or bipolar disorder. Some of them provide social and cognitive remediation programs.

The referral rehabilitation hospitals are chiefly dedicated to traumatic brain injury patients and young patients with anoxic damage, stroke, multiple sclerosis, encephalitis, and postneurosurgery.

The outpatient rehabilitation clinics are largely run by rehabilitation medicine specialists and dedicated to motor rehabilitation of neurologic diseases, but they are developing a growing interest for cognitive rehabilitation.

The developmental clinics in pediatric departments are primarily concerned with early detection of motor and mental delays, and psychosocial interventions, a few of them having specialized human resources dedicated to cognitive rehabilitation.

#### Community Services, Supervised by Allied Health Professionals Including Psychologists, Occupational Therapists, Social Workers, or Rehabilitation Nurses

The community day centers and residential facilities dedicated to neurodegenerative diseases and providing cognitive care are mainly focused in cognitive stimulation and training of activities of daily living.

The community day centers and residential services are dedicated to children and adults with cerebral palsy and other inborn causes of intellectual disability.

#### Community Services Related With the Educational System, Not Included in the Health System

There are psychology and special education services at schools of the National Ministry of Education. There are also study centers dedicated to the compensation of learning difficulties. Additionally, there are adult and senior learning services.

#### Academic Centers Dedicated to Basic and Clinical Neurosciences

These centers are generally in partnership with institutions from the above categories.

### Patient Care Limitations

In spite of the variety of services, patient access to care is limited by several important factors: (1) the location of patients’ home (urban vs suburban or rural), (2) socioeconomic status, (3) mobility, and (4) the level of education of patients and families [26,27]. Furthermore, National Health Service standards of care do not include global access to cognitive interventions [29]. This leads to great heterogeneity on the level of service available, and the type of providers (private vs governmental) between regions [28]. The standards of professional care and practices, certification and training, and how those standards are maintained over time are also not perfectly established [27,28]. Outside of hospitals or other medical institutions, the clinical responsibility for cognitive interventions or local multi-disciplinary teams’ coordination is difficult to understand solely based on professional certification and specialized training [26,28,29].

### Promoter Center Setting

The clinical center where the initial research and development of COGWEB took place was an outpatient memory clinic. This was based in a neurology department in a tertiary hospital that provided care to 400,000 inhabitants. The resident clinical staff included neurologists and neuropsychologists. Patients with suspected cognitive deficits, irrespective of their cause, were referred to this clinic for diagnosis and rehabilitation by other neurologists, neurosurgeons, psychiatrists, rehabilitation medicine physicians, pediatricians, internists, or general practitioners [23].

### Development and Main Functionalities of COGWEB

The COGWEB system is a Web-based working tool that allows for the implementation of personalized cognitive training programs remotely, in the hospital, or patient’s living environment, under continuous supervision by experienced neuropsychologists [24]. Its development started in 2005, and the first clinical center initiated its use in 2007 (promoter center). Then, the system underwent a five-year period of further technological development, refinement, and thorough clinical testing [24]. Over the last three years, this Web-based cognitive training system was integrated into regular clinical practice at the promoter center. This option led to a threefold increase in patient access to supervised cognitive training and, on average, a sevenfold increase in rehabilitation training time, while maintaining human resources expenditures [23]. More recently, a cohort study provided data on patient adherence and intensity of training obtained using this instrument over long periods of time in a common outpatient memory clinic setting [25]. The version used for this study was composed of 30 independent exercises in a computerized game format. They were developed to train various degrees of impairments in specific cognitive domains, such as attention, executive functions, memory, language, praxis, gnosis, and calculus [23,24]. The training sessions were individually prescribed on the Internet by a therapist, just after thorough cognitive assessment and according to personalized plans discussed face-to-face with each patient, as previously described [25]. Internet activities performed by the patients were summarized in several progress graphs (eg, right answers vs wrong answers, levels completed, global training time, or accesses) that were revised weekly by the professional in charge. This information was used to monitor patient’s evolution, as well as to elaborate progress reports or to aid motivation [23,24].

### Network Implementation Procedures

In March 2012, the most important clinical actors and institutions in the field of cognitive impairment assessment, diagnosis, and treatment in Portugal were invited to join the COGWEB network. The institutions included psychiatry, neurology, and rehabilitation medicine departments, as well as more specialized units within these structures like memory and dementia clinics, schizophrenia clinics, day hospitals, and residential facilities. At the time two national workshop meetings were organized to present the COGWEB system and the results of the first clinical studies. Additionally, actors were invited to talk about their clinical settings and difficulties to implement cognitive intervention programs in everyday practice. During the meetings all were allowed to experiment with the COGWEB system, and were formally invited to participate in a collaborative network, due to start in the near future, and with the main purposes of: (1) democratize patient access to specialized Web-based cognitive stimulation, training, or rehabilitation services; (2) putting Web-based cognitive intervention knowledge into routine practice; (3) further develop and tailor the COGWEB system to the needs and requirements of all professionals that use it in their clinical settings, and patients in their communities; (4) foster multi-center research studies in the field of cognitive rehabilitation; and (5) create the environment necessary to foster translational pathways in the field of cognitive neuroscience. The centers that initially accepted to participate in the network were considered as the baseline group. As the network operated as an open system, all centers that joined thereafter were considered new centers for the analysis.

### Network Maintenance Procedures

All centers that decided to adopt the COGWEB system were visited in person by the network founders (VTC and JP), and received the COGWEB training manuals and in-house formation on how to use the system [23,30]. The first visit had an average duration of 2 hours, and included a session with all the clinical staff enrolled in activities with patients having cognitive deficits (eg, physicians, psychologists, therapists, and nurses). This was followed by a practical workshop with the local responsible neuropsychologist and other team staff such as therapists. During this visit, a second encounter was scheduled to discuss the treatment plans of the first patients to enroll in Web-based cognitive training activities.

The final decision to include patients was the responsibility of the local professionals that selected who could benefit the most from the Web-based cognitive training. There were no restrictions related with medical diagnosis or severity of deficits.

Between visits, all centers were regularly updated on new functionalities of the system (eg, an automatic report tool, performance and assiduity alerts, tutorial videos, and Internet manual), availability of new cognitive training exercises (number went from 17 to 34 during the first year of functioning), the results of quality assessment questionnaires to patients and caregivers, and the results of research study protocols and scientific presentations at national and international meetings. This information was passed in newsletter format by email to the local responsible, and also in part diffused in the blog at the project Web page [22], and at the Facebook page. To incorporate professionals’ points-of-view toward the COGWEB system, these actors were challenged to fill opinion Web-questionnaires using Google Docs. The founders’ efforts to improve quality of use of the system by the professionals in active centers included regular in person visits or webinars using Skype and Google Hangouts to discuss patients and methods, with the centers that were comfortable with this type of communication. Web presentations were also used (eg, good practice advice on how to program daily sessions, information on how to use COGWEB materials in exercise book format, and clinical vignettes).

### Ethical Issues

All professionals signed a specific written informed consent. All patients and caregivers also provided written informed consent. This study was approved by the hospital review board and local ethics commission at Hospital São Sebastião, Centro Hospitalar de Entre o Douro e Vouga, Santa Maria da Feira, Portugal (chair, Rui Carrapato, MD, PhD) and Portuguese National Data Protection Commission.

### Financial Issues

Each center that was enrolled in the COGWEB network paid an annual fee to cover training costs, materials, and development of the system. These fees were supported by the centers themselves, research funding, or by third party sponsors listed in the Conflicts of Interest section. The average cost of using the system amounted to US $8.05 per patient and per month (taxes included). Human resources to manage the system locally were the responsibility of the centers.

### Study Flow

**Figure 1:**
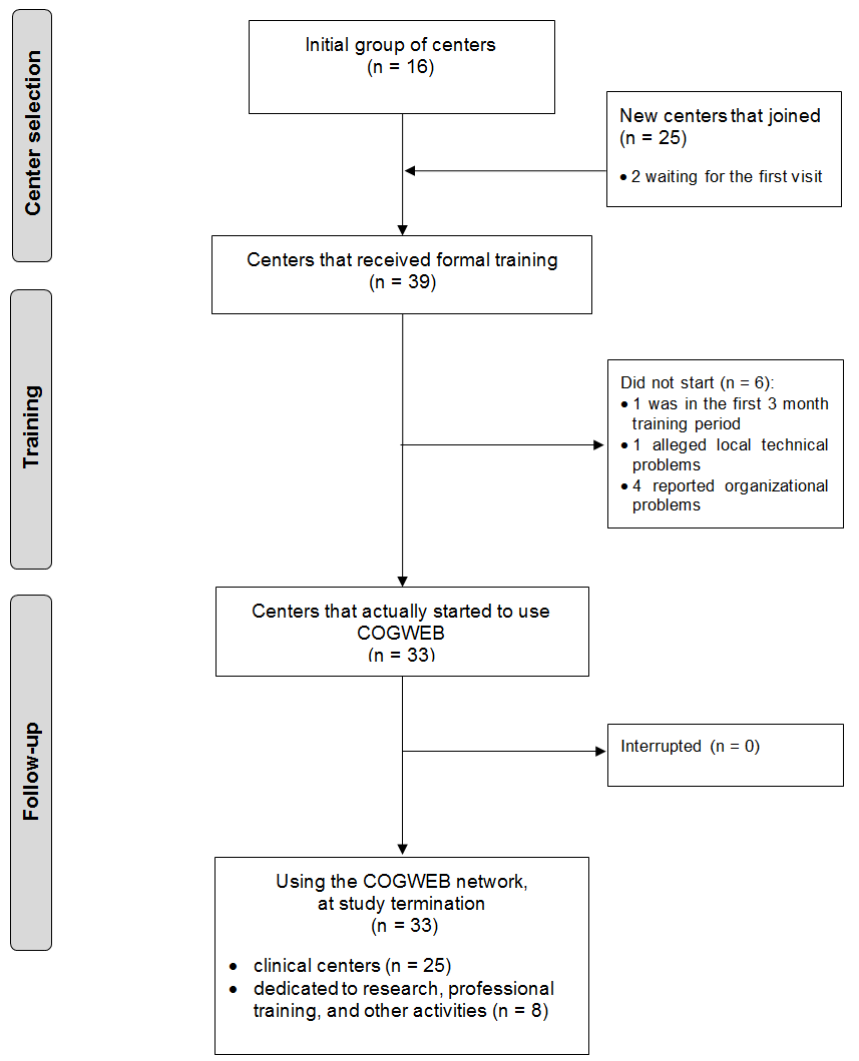
Study flowchart.

There were 68 professionals from 41 centers that received formal training on the COGWEB system during the first year of functioning of the COGWEB network (Figure 1 shows this). The network behavior of these centers was analyzed between August 2012 and September 2013, according to the variables defined for the study.

### Outcomes Definition and Analysis

To evaluate the network as a whole, the centers included were classified as clinical centers, if they were primarily dedicated to clinical activities, or nonclinical centers, if they were focused in research, professional training, and other activities. Additionally, all centers were classified according to the overall services they provided and positioning on the national mental health system setting (Table 1). The number and type of new centers and professionals that joined during the first year of implementation were the elements used to assess the network growth and degree of diversity.

For the subset of the network primarily concerned with clinical activities, the following outcomes were used: (1) number of patients enrolled in Web-based cognitive training activities; (2) number of new patients enrolled per month; (3) characteristics of the patients enrolled (age, gender, level of education, profession, and medical diagnosis); (4) time to start enrolling patients after initial training visit (months); and (5) number of active clinical centers after 1 year, defined as those centers that have patients under treatment at 1 year.

The outcomes (1) and (2) evaluated clinical network growth and the impact on patient access to cognitive treatments. Linear regression was used to identify any time trend in the number of new patients recruited per month. The outcome (3) was concerned with characterization of patient profiles at the centers, and used to compare the profile of the patients enrolled in the first clinical center (promoter) with that in other centers of the network primarily focused in clinical activities. This comparison was used to assess the global impact of the COGWEB network on the diversity of patients (spectra of age and level of education) and diseases offered supervised Web-based cognitive training. This analysis was performed using Student’s *t* test, chi-square, or Fisher’s exact tests.

Finally, the outcomes (4) and (5), combined with outcome (2) were used to obtain knowledge on operative network functioning and long-term sustainability. The median time to start enrolling patients was compared among type of center using the Wilcoxon rank test. All the statistical analysis was performed using the SPSS 20.0 statistical package, considering an alpha = 0.05.

## Results

### Characteristics of the Baseline Centers

The network was initiated in August 2012 with a membership of 16 institutions and 29 health professionals willing to integrate the COGWEB system in their routine (Table 1). These professionals were mainly neuropsychologists and psychologists; two were occupational therapists. The initial centers were all hospital-based clinics, 14 inserted in neurology or psychiatry departments, one in a rehabilitation medicine department, and another in research academic facilities next to a large tertiary center.

**Table 1:** Major types of centers in the network at baseline and 1 year of follow-up (number of centers, trained professionals, and patients enrolled per major category of center).

		Baseline		1 year		
Centers		Centers	Professionals	Centers	Professionals	Patients enrolled
**Clinical**						
	1. Outpatient clinics in neurology or psychiatry hospital departments^b^	14	25	19	38	209
	2. Outpatient clinics in rehabilitation hospital departments^b^	1	2	1	2	2
	3. Outpatient clinics in pediatric hospital departments^b^	-	-	1	1	^a^
	4. Community day care^c^	-	-	2	3	10
	5. Community private practices run by neuropsychologists^c^	-	-	8	8	42
	6. Occupational psychology practice in a major company^c^	-	-	1	1	15
	7. Psychology office at a second grade school^c^	-	-	1	1	20
Subtotal		15	27	33	54	298
**Nonclinical**						
	8. Academic clinical research^d^	1	2	3	8	163
	9. Academic basic research^d^	-	-	1	2	20
	10. Postgraduate professional training^d^	-	-	1	1	NA^e^
	11. Adult learning institutes^c^	-	-	3	3	60
Subtotal		1	2	8	14	243
Combined total		16	29	41	68	541

^a^The single center in this category was waiting for the initial training visit at the end of study.

^b^Hospital-based

^c^Community-based

^d^Academic/education-based

^e^NA = Not applicable

### Characteristics of the Professionals and Centers at 1 Year of Network Functioning

The number of professionals that received specialized training within the network went from 29 to 68 (60 psychologists or neuropsychologists, 4 occupational therapists, 2 neurology residents, 1 psychiatrist, and 1 neurosciences researcher). The mean age of the professionals was 38.1 years (SD 8.8), 83% (57/68) female.

During the first 12 months of functioning, 25 additional centers joined the COGWEB network, from 16 at baseline. There are two of the new centers that have recently joined and were waiting to receive formal training. A total of 41 centers were part of the final analysis. Furthermore, 33 of these centers were classified as clinical (33/41, 80%), while 8 were considered nonclinical and focused in academic research, postgraduate training, or stimulation of normal adults (8/41, 19%) (Table 1).

Considering the services provided by the 25 new centers, 7 belonged to 2 of the initial existing categories (outpatient clinics in neurology or psychiatry departments and academic clinical research centers), and 18 represented 8 new categories of centers (Table 1). At one year, there were 11 different types of centers that could be additionally grouped by major sector of activity as; hospital-based (21/41, 51%), community-based (15/41, 36%), or academic/education-based (5/41, 12%).

From the 39 centers that received training by the end of the study period, 33 (84%) started to use COGWEB, either developing clinical or research activities. Taking into account all the active centers, the median time from the first on-site training visit to the enrollment of the first patient was 1.5 months (interquartile range, 0.5-3.0; SD 1.08 months; 95% CI 1.33-2.15) without differences between types of center (*P*=.57). Among all clinical centers that received formal training (n=31), by the end of the study period, 80% (25/31; n=25) remained actively enrolling patients and using COGWEB. The 6 clinical centers that were not active at the end of the study (6/31, 19%), never started to enroll patients after their first visit; 1 center was in the first 3 month training period (1/6, 16%), 4 reported organizational and local human resources problems (4/6, 66%), and 1 alleged major technical problems (1/6, 16%). All of the centers that started to use COGWEB with their patients (n=25) were active at the end of the 12 months follow-up period, with no dropouts.

### Characteristics of Patients that Received Treatment in Clinical Centers

Among all the 25 clinical centers that started to use the COGWEB system in their activities, a total of 298 patients were enrolled for cognitive training during the first year. The average age was 54.4 years (SD 18.7), 45.3% (135/298; n=135) were female. The patients had diverse formal educational levels, 22.5% (67/298; n=67) from 1-4 years, 28.5% (85/298; n=85) from 5-9 years, 24.8% (74/298; n=74) from 10-12 years, and 24.1% (72/298; n=72) with more than 12 years of school (Table 2). The major causes for cognitive impairment of all the patients treated were; neurodegenerative diseases (115/298, 38.5%; n=115), static structural brain lesions (63/298, 21.1%; n=63), multiple sclerosis and other immune diseases (40/298, 13.4%; n=40), schizophrenia (30/298, 10.0%; n=30), cognitive dysfunction of functional nature (28/298, 9.3%; n=28), attention deficit hyperactivity disorder (12/298, 4.0%; n=12), and others (10/298, 3.3%; n=10) (Table 2).

**Figure 2:**
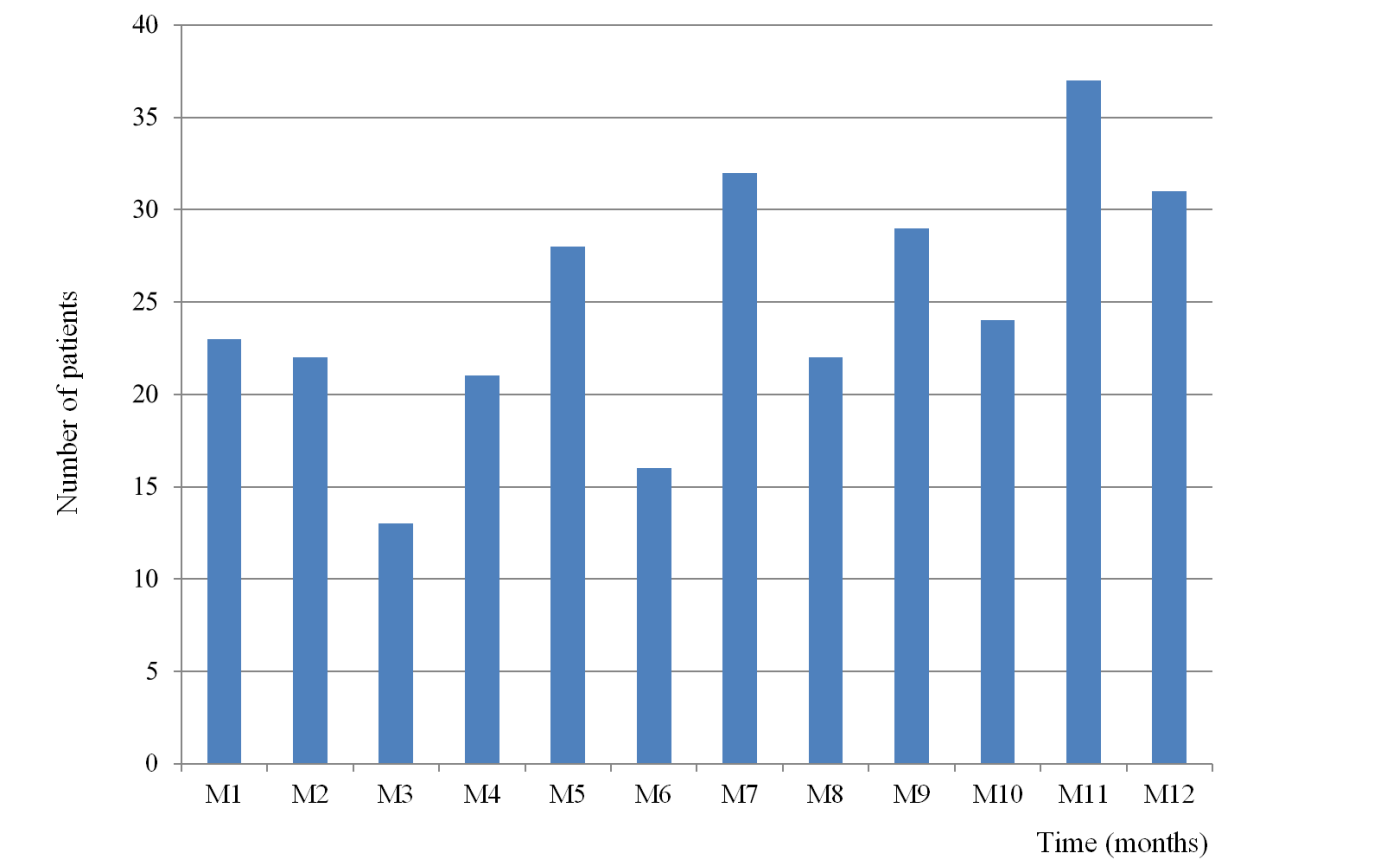
Number of patients enrolled each month in Web-based cognitive training through the COGWEB network.

**Figure 3:**
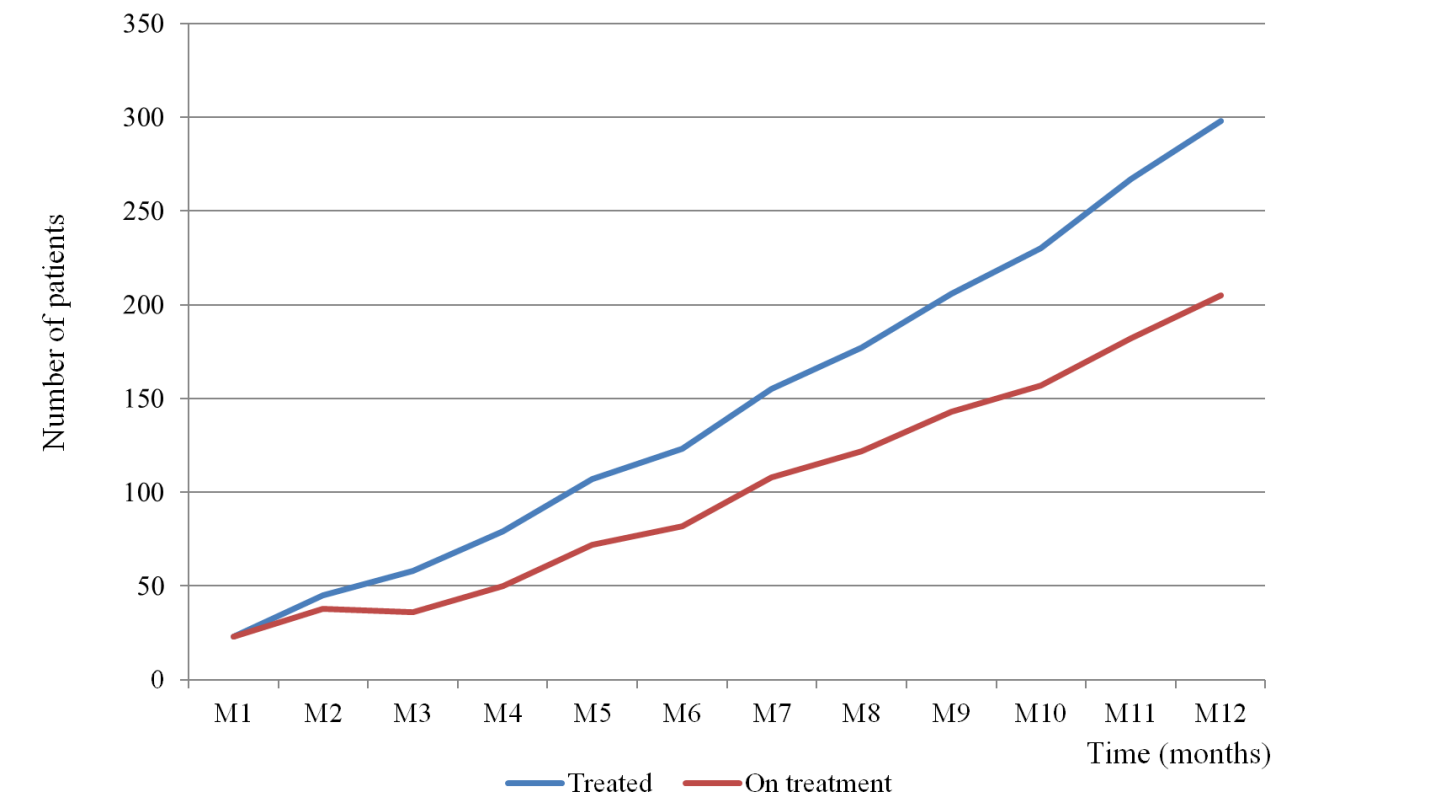
Cumulative number of patients treated during the first year (blue) against the number of patients receiving active treatment trough the COGWEB network each month (red).

During the follow-up period there was a significant increase of the number of patients enrolled every month at the clinical network (*r*=0.6; *P=* .031) (Figure 2 shows this). At 12 months, 205 patients remained on active treatment (Figure 3 show this).

**Table 2:** Description of the patients enrolled at promoter center, other clinical centers, and global clinical network.

		Promoter center	Other clinical centers	Global clinical network
Number of patients		117	181	298
Age, years, average (SD)		45.8 (14.7)	60.1 (19.7)	54.4 (18.7)
**Gender**				
	Female frequency, n (%)	39/117 (33.3)	96/181 (53.0)	135/298 (45.3)
Education, years, average (SD)		8.9 (4.2)	10.6 (5.1)	9.8 (4.8)
**Cause of cognitive impairment, n (%)**				
	Neurodegenerative diseases with dementia	20/117 (17.1)	95/181 (52.4)	115/298 (38.6)
	Stroke, TBI^a^, and other static structural lesions	23/117 (19.7)	40/181 (22.1)	63/298 (21.1)
	Multiple sclerosis and other autoimmune diseases	35/117 (29.9)	5/181 (2.8)	40/298 (13.4)
	Cognitive dysfunction of functional nature	10/117 (8.5)	18/181 (9.9)	28/298 (9.4)
	Schizophrenia	27/117 (23.0)	3/181 (1.7)	30/298 (10.1)
	ADHD^b^	1/117 (0.9)	11/181 (6.1)	12/298 (4.0)
	Others	1/117 (0.9)	9/181 (5.0)	10/298 (3.4)

^a^TBI = traumatic brain injury

^b^ADHD = attention deficit hyperactivity disorder

### Comparison of the First Clinical Center Activity With the Other Network Centers

In Table 2, the patients at the promoter center are compared with the patients at the remaining network, namely: (1) mean age, (2) gender, (3) level of education, and (4) cause of cognitive impairment. The patients recruited at the new network centers were older (*P*<.001). Nonetheless, the new centers also doubled the proportion of patients with less than 20 years of age 5.6% (10/181) versus 2.6% (3/117) at the promoter center. There was a significant difference in the gender distribution (*P*=.01), with more males in the promoter center. The patients’ educational attainment was higher in the new centers than in the promoter (*P*=.005). Considering the distribution of the causes of cognitive impairment, the promoter center enrolled relatively more patients with schizophrenia 23.0% (27/117) versus 1.7% (3/181), *P*<.001, and autoimmune diseases 29.9% (35/117) versus 2.8% (5/181), *P*<.001. Patients with neurodegenerative diseases were the majority of patients enrolled at the new centers (95/181, 52.4%), while their percentage at the promoter center was 17.0% (20/117; *P*<.001). The new centers also enrolled relatively more patients with ADHD, 6.1% (11/181) versus 0.9% (1/117; *P*=.04).

### General Description of Activities at Research Centers

Besides the research and development activities occurring at the promoter center, four academic research centers (three clinical and one basic science) participated in the network, using COGWEB in their studies. These centers were dedicated to the study of the effects of cognitive training across several disease models and settings, and looking for molecular, brain imaging, or neuropsychological biomarkers and characterization of neuroplastic processes. Some of the disease models included Alzheimer’s dementia, schizophrenia, multiple sclerosis, stroke, and school age learning disabilities. A center was dedicated to epidemiological and public health cohort studies. The total number of patients enrolled in all these research activities during the follow-up period amounted to 417, with 183 (43.9%) coming from studies originating outside the promoter center (Table 1).

## Discussion

### Principal Findings

Starting from an initial clinical promoter center, integrated in a wider national mental health system setting in Western Europe, it was possible to implement over a 12 month period a collaborative network composed of 41 centers and 68 professionals. This network was dedicated to cognitive intervention and, for its establishment, took advantage of an innovative Web-based cognitive training system, COGWEB [23,24,30]. This tool was developed for clinical and research purposes at the promoter center, and had proved to be proficient in increasing patient access to care and intensity of cognitive training [23-25]. The process of training and sharing a new working tool, and methods, in the field of cognitive training was the cornerstone for the construction of the COGWEB network, and fostered synergies and cooperation between so diverse centers and settings. Health care is a collaborative endeavor, but the degree of collaboration and exchange depends largely on the ability to share and the reciprocity perceived by all the players and stakeholders of a network [10].

The 16 baseline centers that started the network were all based on hospital institutions. Nonetheless, during the first year of functioning, the network was able to attract 25 new centers, and at the end of the study period 11 different categories of centers were identified (Table 1), with 36% (15/41) of them being primarily based on the community. The diversity of centers and institutions enrolled went from referral hospitals and academic centers to day care institutions, schools, adult learning institutes, and companies. All this variety provided us with a wider view on global patient needs, settings, and professional groups interested in improving their standards of care in the field of cognitive intervention. Considering the main characteristics of the national mental health service where the study occurred, namely the range of environments and existing barriers to patient access to cognitive interventions [28,29], this was an important achievement. Only through an inclusive approach is it possible to enhance solutions within a network environment and bridge the gaps between so diverse settings and professionals like those from referral hospital centers, basic and clinical academic centers, or community based institutions [1,8-10]. The needs for cognitive training in the population are very widespread and growing, mostly due to the multiplicity of diseases associated with cognitive deficits, the wide spectrum of ages of onset, and ageing trends in the population [15,27,29]. Altogether, if the aim is a public health impact in the near future, the multiplicity of solutions and settings connected through a cognitive care collaborative network are an important solution to match current and future needs of the population, at the same time improving the sustainability of health services [2,13].

Although the implementation of the clinical network was only a short period of time, the number of patients provided Web-based cognitive training through the network increased steadily, amounting to more than 30 new patients per month in the last two months. Furthermore, the percentage of patients remaining under clinical supervision at the end of the study period was also high (205/298, 68.8%). These multi-center adherence estimates, during a 12 months follow up, may be comparable with adherence data obtained in a previous cohort study at the promoter center (82.8% at 6 months) [25]. Although an indirect quality measure, the reproduction of the adherence data in this study supports the strategy used for the professionals’ training at the new centers.

The comparison of the characteristics of patients treated at the promoter center with those enrolled at other centers in the clinical network showed a marked increase, with significant differences, in the diversity of diagnosis, spectra of ages, and education. These findings are in accordance with the different categories of centers and types of services provided within the wider mental health system context [26,29]. The achievement of such a variety of settings and diseases is an important characteristic of the clinical network, namely for the implementation of future research studies and tailoring of the COGWEB system to professional and patient needs. A striking finding was the increase in the number and percentage of patients with neurodegenerative diseases (Table 2), possibly in association with the characteristics of the new centers that adhered to the network, with a great proportion being dedicated to neurodegenerative diseases and elder patients (Table 1). This fact probably reflects the distribution of cognitive impairment in an aging population [31], and the willingness of those centers and professionals to adhere to a network dedicated to Internet cognitive training activities [25].

The strategy defined for professional training, network implementation, and maintenance allowed for a median time to start using the COGWEB system in clinical activities of 1.5 months, with 80% (33/41) of the clinical centers active at 12 months and no dropouts. Nonetheless, 4 institutions reported local organizational and human resources restrictions as reasons for not starting to use the system. These estimates are important for programing further network expansion, anticipating points of tension between individual and organizational goals, guaranteeing its alignment with financial incentives, and sustainability [9].

Besides clinical activities, it was verified a remarkable growth in research activities over the network. This finding is of utmost importance because studies originating outside the leading promoter center already represented 43.9% (183/417) of patients enrolled in these activities. Research activity is one of the main purposes of this network, and tightly linked to the capacity to generate innovation, processes, and finally patient outcomes [11,32]. This happens in close resemblance with the development of translational research and translational networks in the fields of oncology [6], pediatrics [33], genetics [34], neurodegenerative diseases [35], virology [36], pharmacology [37], big data bioinformatics [38], epidemiology [39], and public health [32], all good examples of the growing efforts being made to fill the gap and speed processes between basic research and clinical outcomes for communities [11].

### Limitations

The main limitations of this study are related with the youth nature of the COGWEB network (first year of functioning), being difficult to validate the long term sustainability, outcomes, and impact of the network structure. The differences between center characteristics (41 centers distributed by 11 categories), and the small relative number of patients enrolled at each center prevented us from analyzing patient profiles per type of center and establish comparisons. The aggregation of clinical centers into promoter and others was thus necessary. Data on the severity of patient deficits as well as type, intensity, and quality of cognitive training provided were not analyzed. Additional studies are necessary to evaluate the long term impact of the network on global access of patients to supervised cognitive training at the level of the national health system, quality of care provided, and patient outcomes according to major cause of cognitive impairment. Furthermore, the professional members of the network were not addressed directly through a network survey, nor are data available on key players, ties (indegrees and outdegrees), brokers, or sociograms [6]. These points are very important for translational network analysis, and will be addressed in forthcoming studies on the COGWEB network functioning.

### Conclusions

This paper provides insight on the implementation and early outcomes of a large scale multi-organizational cognitive rehabilitation network in a Western European health system environment. Over its first year, there was a major increase in the number, as well as in the clinical diversity, of patients treated and centers, crucial factors for its long term viability. At the beginning of the big data analysis era for neurosciences [40], the consolidation of such a national collaborative network represents an innovative step in mental health care evolution. Furthermore, it may contribute to translational processes in the field of cognitive training and cognitive care, this way providing the foundations for continued innovation, clinical care improvement, and reducing the burden of disease.

## Abbreviations

COGWEB: Web-based cognitive training system
